# Transarterial Embolization for the Treatment of Symptomatic Hip Osteoarthritis

**DOI:** 10.1007/s00270-026-04448-w

**Published:** 2026-04-23

**Authors:** Florian Nima Fleckenstein, Tazio Maleitzke, Stephan Oehme, Stefanie Donner, Timo Alexander Auer, Alexander Hildebrandt, Carsten Perka, Jens Vogel-Claussen, Bernhard Gebauer, Tobias Winkler, Federico Collettini

**Affiliations:** 1 Department of Diagnostic and Interventional Radiology, Corporate Member of Freie Universität Berlin and Humboldt-Universität zu Berlin, Charité – Universitätsmedizin Berlin, Charité Campus Mitte (CCM), Charitéplatz 1, 10117 Berlin, Germany; 2 Berlin Institute of Health (BIH), Charité – Universitätsmedizin Berlin, Anna-Louisa-Karsch 2, 10178 Berlin, Germany; 3 Center for Musculoskeletal Surgery, Corporate Member of Freie Universität Berlin and Humboldt-Universität zu Berlin, Charité – Universitätsmedizin Berlin, Berlin, Germany; 4 Julius Wolff Institute, Berlin Institute of Health at Charité – Universitätsmedizin Berlin, Berlin, Germany; 5 Trauma Orthopaedic Research Copenhagen Hvidovre (TORCH), Department of Orthopaedic Surgery, Copenhagen University Hospital - Amager and Hvidovre, Hvidovre, Denmark; 6 Department of Clinical Medicine, University of Copenhagen, Copenhagen, Denmark; 7 BIH Center for Regenerative Therapies, Berlin Institute of Health at Charité – Universitätsmedizin Berlin, Berlin, Germany

## Abstract

**Purpose:**

To evaluate the safety and clinical effectiveness of transarterial embolization (TAE) using temporary embolic agents in patients with symptomatic hip osteoarthritis (OA) refractory to conservative treatment.

**Materials and Methods:**

This single-centre retrospective analysis included patients undergoing TAE between January 2023 and April 2025. Eligibility required ≥ 3 months of clinically and radiographically confirmed hip OA unresponsive to conservative therapy. Embolization was performed using rapidly resorbable gelatin microspheres or imipenem/cilastatin. Outcomes included technical success, adverse events, and patient-reported outcomes (numeric rating scale [NRS] pain; Hip Disability and Osteoarthritis Outcome Score [HOOS]) assessed at baseline and up to 12 months. Clinically meaningful improvement was defined using minimum clinically important difference (MCID) thresholds (≥ 2 points for NRS; ≥ 10 points for HOOS).

**Results:**

Forty-one patients (median age 65 years) underwent TAE with a technical success rate of 97.5% (40/41). The lateral circumflex femoral artery was embolized in all cases, with additional internal iliac branches treated in 27.5% (11/40). No major or moderate adverse events occurred; minor transient skin discoloration was observed in 7.3% (all after imipenem/cilastatin). Median NRS decreased from 7 to 4 at 12 months (*p* < .001), with significant improvement across all HOOS domains. MCID was achieved in 64.3% for NRS and 85–92% for HOOS. Two patients (4.9%) underwent total hip replacement.

**Conclusion:**

TAE using temporary embolic agents was associated with sustained pain reduction and functional improvement at 12 months with a favourable safety profile.

**Graphic Abstract:**

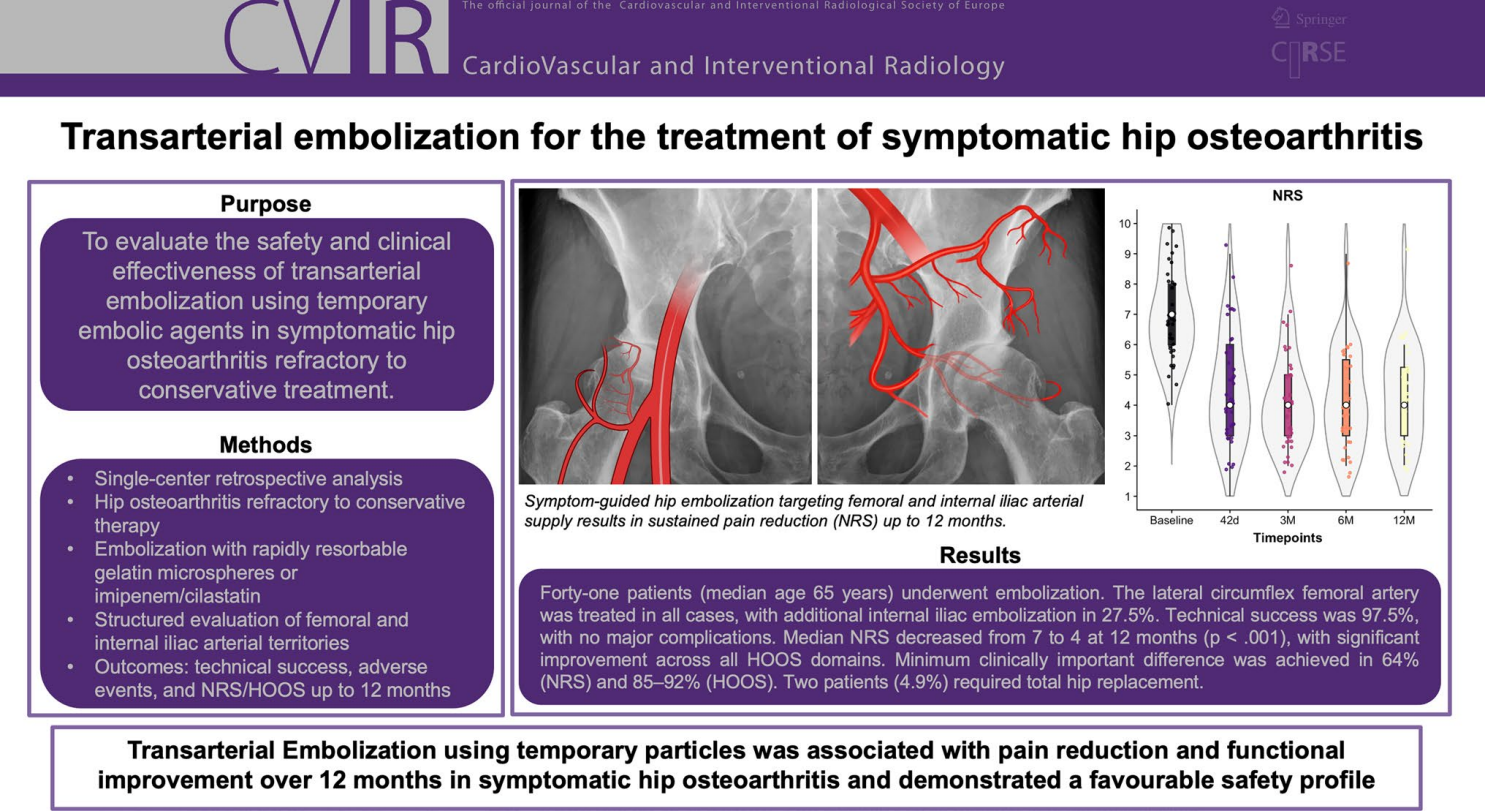

## Introduction

Hip osteoarthritis (OA) is a common and increasingly prevalent cause of pain and functional limitation in middle-aged and older adults, with a lifetime risk of symptomatic disease estimated at one in four individuals [1]. Management of hip OA typically involves non-steroidal anti-inflammatory drugs (NSAIDs), structured physiotherapy programs, and optionally intra-articular corticosteroid injections for short-term symptom relief, while total hip replacement remains the definitive treatment for advanced disease. The lifetime risk of requiring total hip arthroplasty after a diagnosis of hip OA is approximately 14% [2]. Yet, many patients experience persistent pain despite conservative treatment but are not suited for surgery due to comorbidities or age. Thus, there is a need for minimally invasive treatments that target pathological drivers of OA.

Chronic synovial inflammation and pathological neovascularization are key contributors to pain and disability in OA [3]. These neovessels are accompanied by pain-sensitive nerve fibres, linking angiogenesis to pain sensitization [4]. In this context, *transarterial embolization* (TAE) has been proposed as a novel treatment for OA [5–8]. Following encouraging results of genicular artery embolization (GAE) in knee OA, TAE has been applied to the hip joint. Early reports demonstrated significant pain reduction and functional improvement mainly in patients with greater trochanteric pain syndrome (GTPS) [9–11]. More recently, a prospective pilot study on hip OA demonstrated early improvements in pain and function, supporting the feasibility of this approach [12]. These findings are complemented by the *HipE study*, which confirmed sustained clinical benefit at 12 months, with significant clinical improvements and a favourable safety profile in patients with hip OA and/or GTPS [13]. Despite these advances, long-term evidence in homogeneous cohorts of patients with hip OA remains limited, highlighting the need for further studies focusing specifically on this population.

The purpose of this study was to evaluate the safety and efficacy of TAE in patients with symptomatic hip OA refractory to conventional therapy with clinical follow-up available for up to 12 months.

## Materials and Methods

This is a single-centre, retrospective analysis of a prospectively collected database including patients undergoing TAE for hip OA between January 2023 and April 2025. The study was approved by the institutional ethics committee (EA2/096/24), and written informed consent was obtained from all patients. Indications for TAE included: (i) adults with hip pain clinically and radiographically consistent with hip OA, confirmed by weight-bearing radiographs and/or MRI demonstrating OA changes and (ii) refractory to ≥ 3 months of conservative treatment. Key contraindications included fractures, active joint infection, significant peripheral arterial disease and uncorrectable coagulopathy. All patients were evaluated by an orthopaedic surgeon and discussed in a multidisciplinary board before undergoing treatment. Radiographic severity was graded according to the Kellgren–Lawrence (KL) classification.

### Embolization Procedure

**Fig. 1:**
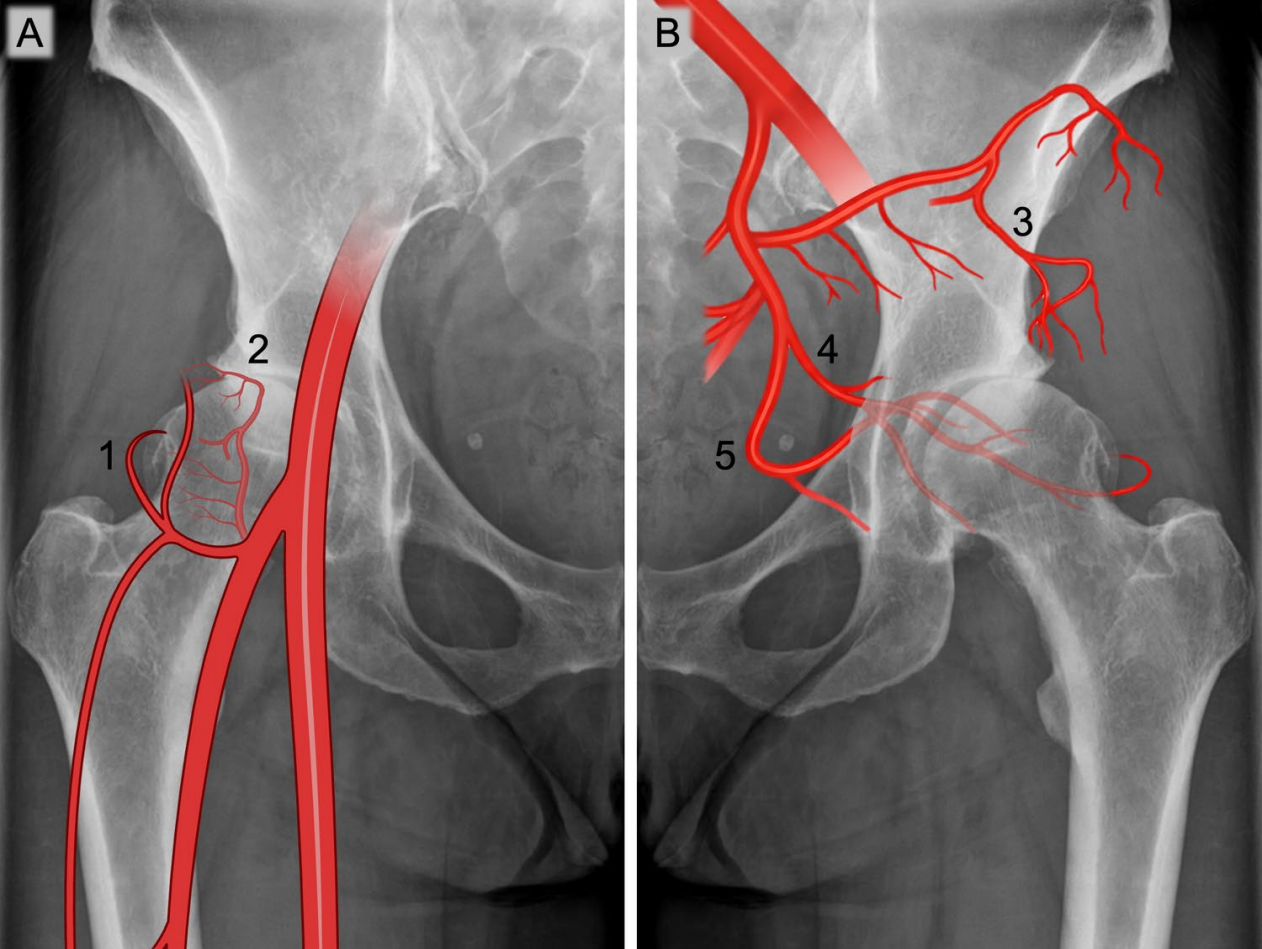
Schematic representation of the arterial supply of the hip joint. **A.** Illustration of the femoral arterial axis demonstrating (1) the lateral circumflex femoral artery and (2) the medial circumflex femoral artery. The lateral circumflex femoral artery primarily supplies the lateral periarticular soft tissues and synovium of the hip joint, whereas the medial circumflex femoral artery contributes predominantly to femoral head perfusion via retinacular branches. **B.** Illustration of the periarticular arterial network highlighting contributions from the internal iliac arterial system. In addition to the femoral arterial supply, multiple branches originating from the internal iliac artery contribute to the vascularization of the hip joint. These include deep branches of the superior gluteal artery (3), the inferior gluteal artery (4), and the obturator artery (5). Created in BioRender. Fleckenstein, FN & Zhou, S. (2026)

**Fig. 2:**
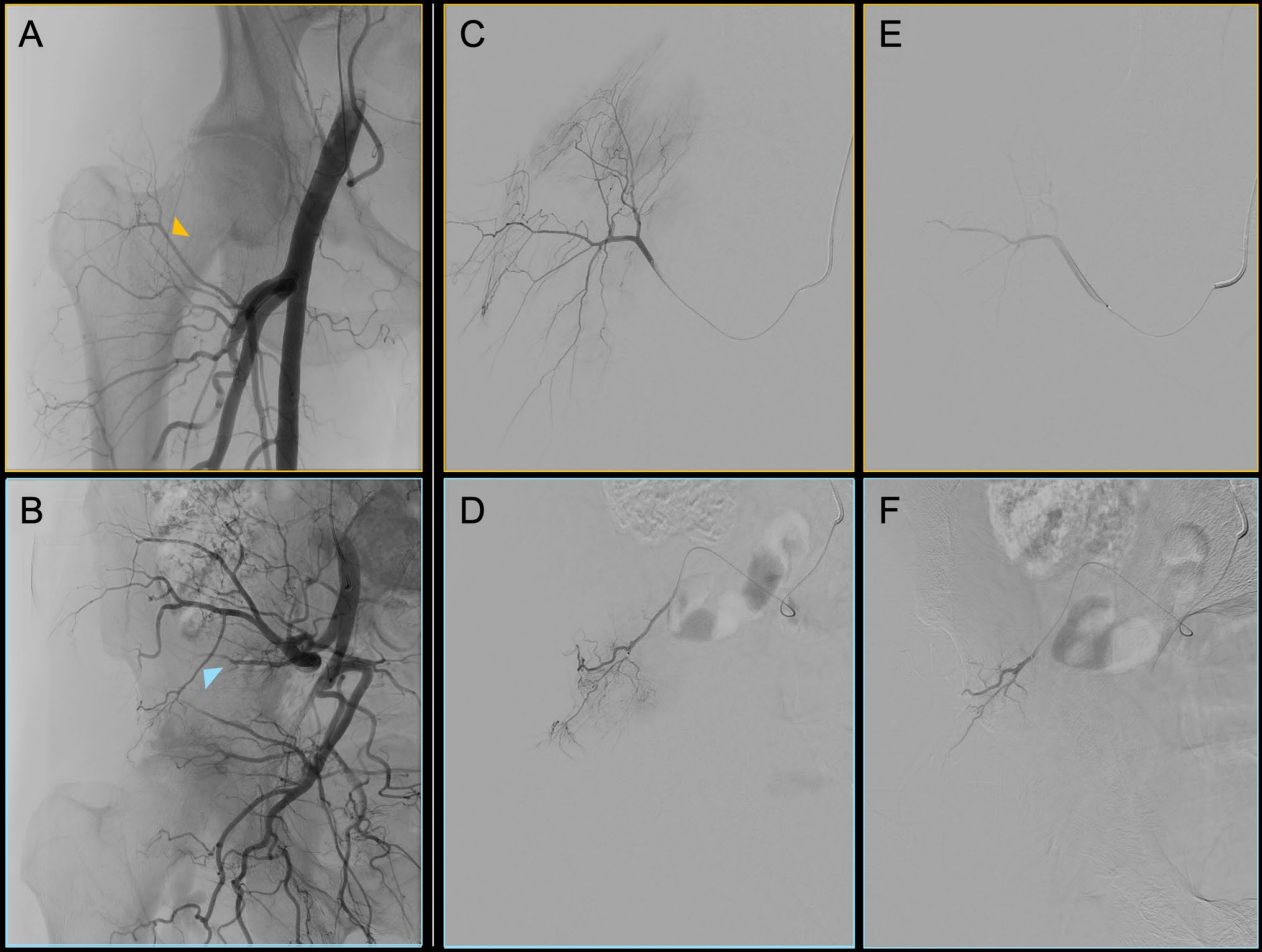
Peri-interventional imaging of the right hip joint in a 72-year-old patient with symptomatic hip osteoarthritis.** A and B.** Angiography of the right common femoral artery (**A**) and the internal iliac artery (**B**) demonstrating the arterial anatomy of the hip. Arrows indicate potential target vessels: lateral circumflex femoral artery (orange); deep branch of the superior gluteal artery (blue). **C and D.** Superselective pre-interventional DSA of the lateral circumflex femoral artery (**C**) and the deep branch of the superior gluteal artery (**D**) showing a pronounced synovial hypervascularity corresponding to the patient’s pain localization, respectively. These hyperaemic patterns reflect pathological neovascularization typically seen in osteoarthritic joints. **E and F.** Superselective post-interventional DSA following targeted embolization of the lateral circumflex femoral artery (**E**) and the deep branch of the superior gluteal artery (**F**) with temporary particles. A complete disappearance of the pathological blush can be seen, accompanied by temporary flow stasis within the treated branches while maintaining perfusion of the proximal arterial segments

All patients were awake during the procedure to allow intra-procedural pain feedback. After application of local anaesthesia, vascular access was obtained under ultrasound guidance via the contralateral common femoral artery. After cross-over manoeuvre, a 4 Fr sheath (65 cm, 4 Fr Fortress; Biotronik) was inserted into the ipsilateral iliac axis. A 4 Fr base catheter (RIM, Rösch Inferior Mesenteric, Cordis; or RBI, Renal Bifurcation, Merit Medical) was used depending on vascular anatomy and operator preference. Angiographic evaluation systematically included branches of the profunda femoris arterial system (Fig. 1A), particularly the lateral circumflex femoral artery, as well as branches of the internal iliac artery (Fig. 1B), including the gluteal and obturator arteries, given their potential contribution to synovial perfusion of the hip joint through periarticular anastomotic networks. Based on these anatomical considerations and clinical experience, pain distribution may correspond to vascular territories, with lateral symptoms often relating to the lateral circumflex femoral artery, posterior symptoms to gluteal branches, and medial or anterior pain to obturator contributions. Accordingly, an initial DSA was performed after positioning the base catheter sequentially within the common femoral (Fig. 2A) and internal iliac arteries (Fig. 2B). For superselective catheterization of potential target vessels a 1.7 Fr microcatheter (Veloute, Asahi Intecc or Progreat Lambda, Terumo) was coaxially advanced over a 0.014″ guidewire (CHIKAI, Asahi Intecc or Fathom, Boston Scientific).

Embolization was performed based on predefined angiographic and clinical criteria. The primary criterion for embolization was the presence of a pathological hyperaemic blush on superselective DSA (Fig. 2C-D). In the absence of a visible blush, embolization was performed when at least two of the following secondary criteria were fulfilled: (i) reproduction of the patient’s characteristic OA-related pain (“evoked pain”) during superselective contrast injection; (ii) anatomical correspondence between the vessel territory and the patient-specific pain distribution as documented on standardized pre-procedural pain mapping; or (iii) angiographic evidence of collateral perfusion supplying a target vessel.

Embolization involved injecting rapidly resorbable gelatin microspheres (RRGM; Nexsphere-F, 100–300 μm, NextBiomedical) or imipenem/cilastatin (IPM/CS; 500 mg/500 mg, Merck). RRGMs were suspended in 10 mL saline, mixed for approximately one minute to ensure uniform dispersion, and subsequently combined with 10 mL iodinated contrast medium prior to injection. IPM/CS was suspended in 10 mL iodinated contrast medium immediately before administration to create a transient particulate suspension. The choice of embolic agent was primarily based on temporal availability, with IPM/CS used initially and RRGM used after its introduction. Final selection was at the discretion of the operator. Endpoint of embolization was defined as: (i) flow stasis on fluoroscopic imaging and (ii) complete or near-complete pruning of the pathological blush on post-embolization DSA (Fig. 2E-F). Participants were admitted overnight for observation and received a clinical examination and groin ultrasound the next day prior to discharge.

Technical success of the procedure was defined as successful catheterization and embolization of the arterial branches feeding the hypervascular synovium in the affected hip, with elimination of abnormal contrast blush on the final DSA.

### Follow-Up and Outcome Measures

Procedure time, defined as the interval from vascular access to sheath removal, as well as the quantity of embolic agent used, were recorded. Patient outcomes were assessed at baseline, six weeks, three months, six months and 12 month following TAE using the numeric rating scale (NRS) for pain and the Hip Disability and Osteoarthritis Outcome Score (HOOS) with five subscores (Pain; Symptoms; Activities of Daily Living (ADL); Sport and Recreation; Quality of Life (QOL)) [14]. Based on prior studies, clinically meaningful change was defined using minimum clinically important difference (MCID) thresholds of ≥ 2 points for NRS pain and ≥ 10 points for HOOS subscores. The HOOS threshold of 10 points corresponds approximately to half a standard deviation, a commonly accepted distribution-based approach for determining MCID in the absence of anchor-based estimates for hip OA, and is consistent with methodology applied in previous embolization studies [15, 16]. Further, any significant escalation in pain management (or transition to invasive therapies) was documented in the follow-up documentation. Embolization-related complications were classified according to the modified CIRSE Classification System for Complications, which categorizes events based on clinical outcome, hospital stay, and procedural success or failure [17].

### Statistical Analysis

All analyses were carried out using Jamovi version 2.3 (The jamovi project, Sydney, Australia). Descriptive statistics were used to summarize NRS pain and HOOS subscores, which were visualized as violin boxplots for each follow-up. Continuous variables are reported as medians with interquartile ranges (IQR), and categorical variables as percentages. Group differences were examined using a nonparametric repeated-measures method (Friedman test), followed by Durbin–Conover pairwise comparisons.

## Results

### Patient Characteristics

A total of 41 patients (20 women; median age 65 years; IQR 59–72 years) underwent TAE for symptomatic hip OA. Table 1 summarizes baseline patients characteristics. Technical success was achieved in 97.5% (40/41). In one patient, TAE was not performed as none of the predefined angiographic criteria were fulfilled. Median procedure time was 41.6 min (IQR 30.1–49.8) with a median dose area product of 4432 µGy*m^2^ (IQR 847–5580). The lateral circumflex femoral artery was embolized in all cases (40/40), with additional embolization of gluteal or obturator branches in 27% (11/40) due to collateral supply of the symptomatic region. RRGMs were applied in 46% (19/40) and IPM/CS was used as the embolic agent in 21 procedures (51%, 21/40). The median embolic volume 16 mL (IQR 12.1–19.5) for RRGMs and 6 mL (IQR, 4.4–8.7) for IPM/CS.

**Table 1:** Patient demographics, procedural details, and adverse events

Baseline characteristics (*n* = 41)	
Median age, years (IQR)	65 years (59–72)
Sex (female/male)	20/21
Median BMI (IQR)	26.4 (22.1–33.5)
KL Grade (%)	
II	2 (4.9)
III	28 (68.3)
IV	11 (26.8)
Procedural details (*n* = 41)	
Technical success (%)	40/41 (97.5)
Treated side, right/left	22/19
Target vessel origin (*n* = 40):	
Lateral circumflex femoral artery (%)	40/40 (100)
Medial circumflex femoral artery (%)	12/40 (30)
Inferior gluteal artery (%)	9/40 (22.5)
Obturator artery (%)	8/40 (20)
Deep branch superior gluteal artery (%)	5/40 (12.5)
Median procedure time (IQR)	41.6 min (30.1–49.8)
Mean dose area product (IQR)	4432 µGy m^2^ (847–5580)
Embolic	
Nexsphere-F (%)	19/40 (47.5)
Median volume of embolic injected (IQR)	16 mL (12.1–19.5)
Imipenem/Cilastatin (%)	21/40 (52.5)
Median volume of embolic injected (IQR)	6 mL (4.4–8.7)
Adverse events	
Grade 1 (minor, no therapy, no sequelae) (%)	3/41 (7.3)
Grade 2 (minor, nominal therapy, no sequelae) (%)	0/41 (0)
Grade 3–6 (major, requiring therapy or death) (%)	0/41 (0)

*KL Kellgren***–***Lawrence; BMI Body mass index; IQR Interquartile range*

### Follow-Up and Patient-Reported Outcomes

**Fig. 3:**
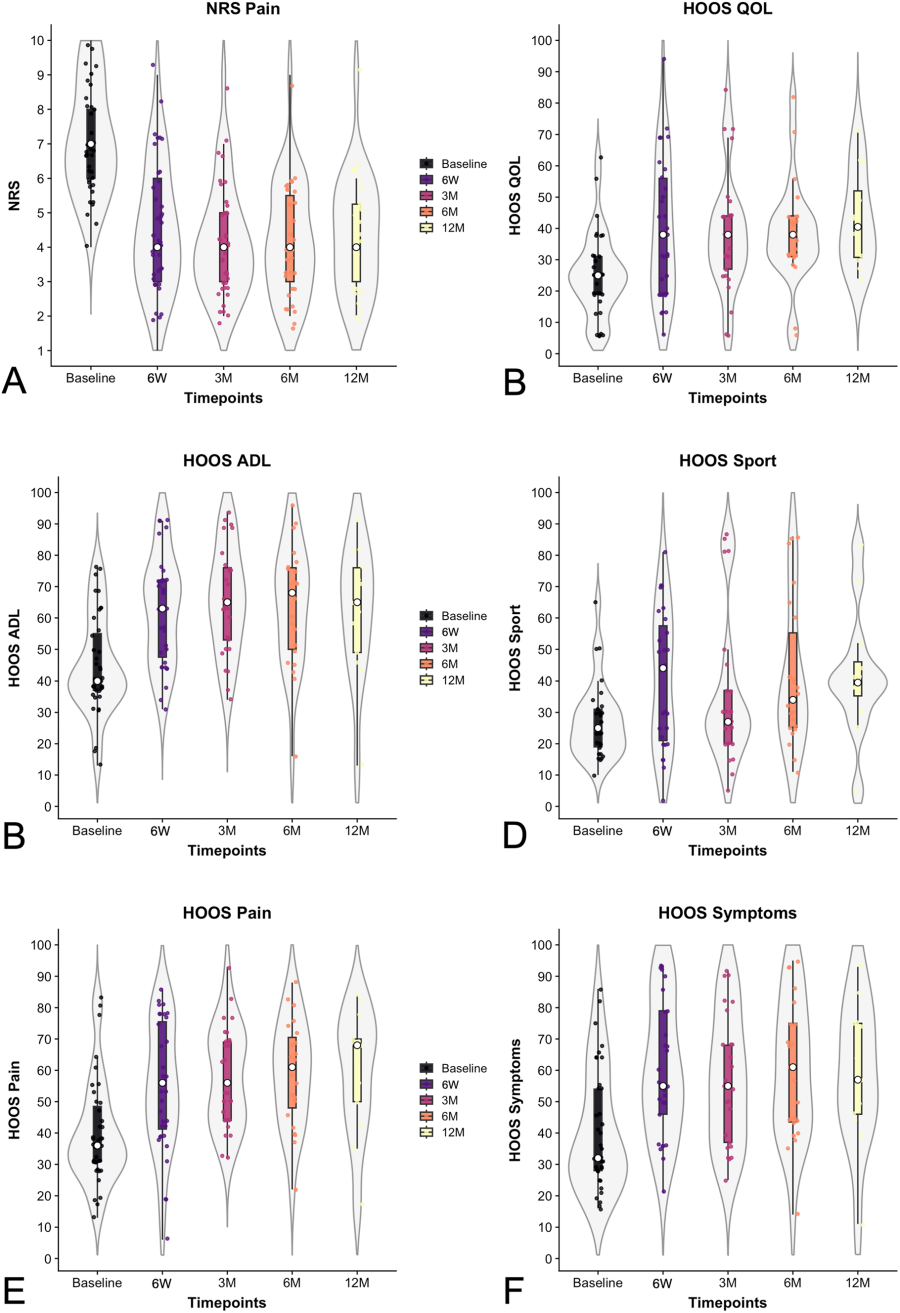
Violin boxplots with median values for NRS pain and HOOS subscores over time. A total of 41 patients were included. Follow-up was available for 97.5% (*n* = 40) at 6 weeks, 97.5% (*n* = 40) at 3 months, and 85.5% (*n* = 35) at 6 months. Twelve-month data were available for 68.5% (*n* = 28). NRS Numeric Rating Scale; HOOS = Hip Disability and Osteoarthritis Outcome Score; QoL quality of life; ADL activities of daily life. Higher HOOS scores indicate improvement; lower NRS scores indicate improvement

Clinical follow-up was available for 98% (40/41) at six weeks, 98% (40/41) at three and 86% (35/41) at six months. Twelve-month follow-up was available for 69% (28/41) of all patients. Figure 3 shows the distribution of patient-reported outcomes at each follow-up. The median baseline NRS pain was 7 (IQR 6–8). At six months, median NRS pain decreased to 4 (IQR 3–5; *p* < 0.001). This was sustained at 12 months with a median NRS pain of 4 (IQR 3–5; *p* < 0.001 (Fig. 3A). The HOOS subscores demonstrated consistent improvements at 12 months compared to baseline. The median HOOS-QOL increased from 25 (IQR 11–30) at baseline to 38 ((IQR 30–49); *p* = 0.027) at 12 months (Fig. 3B) and the HOOS-ADL increased from 40 (IQR 36–54) at baseline to 65 ((IQR 49–76); *p* = 0.034 (Fig. 3C)) at 12 months. The HOOS-Sport increased from 20 at baseline (IQR 11–30) to 38 ((IQR 30–44); *p* < 0.001 (Fig. 3D)) at 12 months. Median HOOS-Pain increased from 36 (IQR 30–48) at baseline to 68 ((IQR 50–70); *p* = 0.004 (Fig. 3E)) at 12 months and the HOOS-Symptoms increased from 32 (IQR 28–54) at baseline to 57 ((IQR 46–75); *p* = 0.028 (Fig. 3F)) at 12 months. For NRS pain, MCID was achieved in 64% at 12 months. Across all HOOS domains, 85–92% of patients achieved MCID at 12 months. Of note, two patients (both KL IV) received a total hip replacement after not-responding to the procedure seven and nine months after TAE. Additionally, no patient reported escalation to invasive pain management strategies during follow-up.

### Adverse Events

No major or moderate adverse events occurred. Minor, self-limiting complications included three patients treated with IPM/CS with transient skin discolorization (7.3%), resolving spontaneously within 24h without sequelae in all patients. No cases of infection, skin- or clinically apparent osteonecrosis were documented.

## Discussion

In this cohort, TAE was demonstrated to be safe and to effectively reduce pain and improve function in patients with symptomatic hip OA refractory to conservative therapy. Median NRS pain decreased from baseline throughout all follow-up timepoints, paralleled by a marked increase in all HOOS subscores. These findings suggest that TAE targeting synovial neovascularization is associated with sustained pain reduction and functional improvement in patients with symptomatic hip OA, while demonstrating a favourable safety profile in this cohort.

The presented results are consistent with and extend the emerging literature on TAE for hip-related pain [9–13]. Correa et al. first reported a proof-of-concept case of lateral circumflex femoral artery embolization in a patient with severe hip synovitis, demonstrating complete pain relief and MRI-confirmed resolution of synovial enhancement at four months [9]. In their subsequent prospective series of 13 patients with hip OA and GTPS, the median visual analogue scale (VAS) decreased from 10 to 2 and the Western Ontario and McMaster Universities Osteoarthritis Index (WOMAC) total from 77 to 27 at six months, without relevant complications [10]. More recently, Giordani et al. reported midterm follow-up results from the *LATINO-Hip registry*, including 31 patients with GTPS treated by lateral circumflex femoral artery embolization, demonstrating safety and sustained pain reduction in the majority of patients [11]. A recent prospective pilot study by Feier et al. further supports the feasibility of hip TAE in OA using RRGMs, demonstrating early improvements in pain and function. Similar to our study, TAE was primarily performed via the lateral circumflex femoral artery. However, follow-up was limited to 12 weeks, thereby restricting conclusions regarding the durability of treatment effects. Prospective data from the *HipE study* further support these findings, with clinical success rates of approximately 70–75% up to 12 months and a favourable safety profile [13]. Notably, these results were observed in a cohort including both hip OA and GTPS, reflecting the shared pathophysiological basis of inflammation and neovascularization.

In contrast to mixed populations, the present study focused exclusively on patients with clinically and radiographically confirmed hip OA, thereby providing data in a more homogeneous population. This distinction is important, as GTPS represents a periarticular tendinopathy with partially different biomechanics and pain generators, which may influence treatment response. Furthermore, a methodological distinction of this study is the systematic evaluation of the entire pelvic arterial circulation in addition to the femoral arterial system. Whenever DSA revealed hyperaemic blushes in the gluteal or obturator territories, superselective embolization was performed in addition to the circumflex femoral arteries. This systematic and comprehensive approach has not been consistently reported in previous reports [9–13]. Given the extensive collateral network between the deep femoral and internal iliac systems, such extended targeting may enhance therapeutic efficacy by eliminating alternative routes of synovial perfusion. This concept parallels observations from GAE. In the Landers et al. randomized trial, post-hoc analysis demonstrated significant better outcomes when multiple genicular territories were embolized compared to single-vessel embolization [18].

The use of temporary embolic agents provides reversible vascular occlusion that allows thrombotic elimination of pathological neovessels while preserving normal tissue perfusion as the material resorbs [19, 20]. This is particularly important in the hip, where the femoral head is supplied almost exclusively by the superior and inferior retinacular arteries arising from the circumflex femoral arteries. These vessels function as end-arterial branches and are vulnerable to perfusion disturbances [21]. Permanent embolics carry a theoretical risk of compromising femoral head perfusion through irreversible occlusion of these branches, potentially leading to osteonecrosis. In our cohort, the safety profile was favourable and comparable to prior musculoskeletal embolization studies, with no ischaemic complications observed [8–13, 15, 17–20, 22]. Notably, no clinically apparent femoral head osteonecrosis or persistent neural injury occurred, supporting the safety of TAE in this vascular territory when performed with careful technique and real-time patient feedback.

Potential differences between temporary embolic agents should also be considered. RRGMs may allow a more controlled and homogeneous delivery compared to IPM/CS, which forms non-calibrated, non-spherical crystals [20]. In addition, Nexsphere-F is CE-marked in Europe and approved for OA-related embolization, providing a standardized treatment option. However, these observations remain hypothesis-generated and require confirmation in comparative studies.

Of note, two patients (4.9%) proceeded to total hip replacement after TAE due to insufficient clinical response. Comparable findings in GAE for knee OA showed that a small proportion (5.2%) of patients ultimately require joint replacement despite embolization [23]. These findings suggest that TAE does not appear to compromise the feasibility of subsequent total joint arthroplasty in non-responders. [24].

Despite these encouraging results, limitations must be acknowledged. This is a retrospective, single-centre study with a moderate sample size without a control arm, which precludes differentiation between true treatment effect and placebo response. Objective imaging follow-up was not performed systematically, so morphological changes such as synovial volume reduction could not be correlated with clinical improvement. In addition, although pain relief persisted up to 12 months, longer-term durability remains to be determined, as revascularization or disease progression may occur beyond one year.

## Conclusion

In this single-centre cohort of patients with symptomatic hip OA, TAE with temporary embolic agents was associated with significant and sustained pain reduction and functional improvement at 12 months. The procedure demonstrated a favourable safety profile, with no major complications observed. Larger controlled studies are warranted to further define the role of TAE in the management of hip OA.

## Abbreviations

ADL: Activities of daily living
AE: Adverse event
BMI: Body mass index
DSA: Digital subtraction angiography
GAE: Genicular artery embolization
HOOS: Hip disability and osteoarthritis outcome score
IQR: Interquartile range
IPM/CS: Imipenem/Cilastatin
KL: Kellgren–Lawrence
MCID: Minimum clinically important difference
NRS: Numeric rating scale
NSAIDs: Non-steroidal anti-inflammatory drugs
OA: Osteoarthritis
QOL: Quality of life
RRGM: Rapidly resorbable gelatin microspheres
TAE: Transarterial embolization
